# Investigation of the molecular switching process between spin crossover states of triazole complexes as basis for optical sensing applications

**DOI:** 10.1038/s41598-024-56427-1

**Published:** 2024-03-11

**Authors:** Axel Günther, Yves Deja, Maximilian Kilic, Kevin Tran, Pavan Kotra, Franz Renz, Wolfgang Kowalsky, Bernhard Roth

**Affiliations:** 1 Hannover Centre for Optical Technologies, Leibniz University of Hannover, 30167 Hannover, Germany; 2 Institute of High Frequency Technology, Technical University Braunschweig, 38106 Braunschweig, Germany; 3 Institute of Inorganic Chemistry, Leibniz University Hannover, 30167 Hannover, Germany; 4 Cluster of Excellence PhoenixD (Photonics, Optics and Engineering-Innovation Across Disciplines), 30167 Hannover, Germany

**Keywords:** Optical sensing, Molecular switches, Optical waveguides, Optical materials and structures, Materials for optics, Optical materials and structures, Materials for optics

## Abstract

With the advent of the first laser sources and suitable detectors, optical sensor applications immediately also came into focus. During the last decades, a huge variety of optical sensor concepts were developed, yet the forecast for the future application potential appears even larger. In this context, the development of new sensor probes at different scales down to the atomic or molecular level open new avenues for research and development. We investigated an iron based triazole molecular spin-crossover complex changing its absorption characteristics significantly by varying environmental parameters such as humidity, temperature, magnetic or electric field, respectively, with respect to its suitability for a new class of versatile molecular sensor probes. Hereby, besides the investigation of synthesized pure bulk material using different analyzing methods, we also studied amorphous micro particles which were applied in or onto optical waveguide structures. We found that significant changes of the reflection spectra can also be obtained after combining the particles with different types of optical waveguides.The obtained results demonstrate the suitability of the material complex for a broad field of future sensor applications.

## Introduction

Optical sensing has become increasingly interesting during the last decades by enabling highly precise manufacturing technologies of optical components by two-photon polymerization (TPP)^1–5^, UV-nanoimprint lithography^6–9^or microscope projection photolithography (MPP)^10–12^, among others. These technologies enabled the fabrication of highly sensitive optical structures for determining humidity and temperature, as single molecule detector or to characterize mechanical influences such as bending, pressure or strain, respectively. Therefore, various structures e.g. ring resonators^13–17^, Bragg gratings^18–20^, Mach-Zehnder interferometers^21–23^ or plasmonic sensors^24–27^, to mention a few of them, were employed. These structures can be highly sensitive and specific, determined by the structure type, feature size and quality, respectively. They enable detection of single nanoparticles^14^, low concentration of viruses^13^ or various organic materials^25^, refractive index changes^23^ or even strain and temperature within harsh conditions^18^, respectively. Depending on the application, the structures might not suited to be used as sensor directly and special materials are required changing their optical characteristics due to varying environmental conditions or in presence of specific molecules^28–30^. Very promising materials in that area are molecular switches^31–33^. They are changing their optical response due to varying environmental conditions. The switching mechanism of the complex that we used in this study is based on a reversible spin transition and is known as the spin crossover (SCO) effect^34^. The SCO can either be induced by physical or chemical external influences^35,36^. In this particular case, the used Iron(II)-complexes are in the low-spin state (LS) at room temperature and exhibit a change of their spin state to the high-spin state (HS). Thereby, these compounds show a characteristic colour change from pink to white^37^. For the used triazole complexes it is known that this can be induced by a change of humidity, temperature and magnetic fields^38^. Other stimuli like low concentrations of volatile organic compounds can also be used to induce this effect. This was also observed for other systems like tetrazole complexes. The spin state switching of tetrazole complexes can, for example, be induced by small amounts of ammonia^39^. Monitoring the state of the molecular switches using optical waveguides as basic structures does not require complex structures. The complex has to interact with the light which requires to mix the molecular switches inside the core material or to apply it outside. Hereby, the usage of polymer waveguides offer a possibility to mix particles inside the core material or apply them onto different types of waveguiding structures, respectively. The most common technique to produce polymer based waveguides, next to the previous mentioned ones, are printing^40–42^, hot embossing^43,44^ or self-writing^45–47^, respectively. Using these techniques enables the fabrication of numerous waveguides with modified particle concentrations in the core. In this work we investigated an iron-triazole complex which can be switched by multiple external parameters enabling an auspicious element to detect changes of various environmental parameters. We investigated in detail the optical response of the bulk material at different environmental conditions, e.g. by changing temperature, humidity or electrical and magnetic fields. Additionally, we mixed the iron complex particles with an transparent epoxy resin which we used as core material for hot embossed waveguides and to fabricate self-written waveguides (SWW). Thus, we were able to place the particles in and on the core of the measurement probes. Hereby, the width of the SWWs depends on the mode field diameter of the incoming beam which starts the writing process of the waveguides core. Furthermore, this allows to change the size of the waveguides to realize polymer based single mode structures.

## Materials

The used Iron(II) Triazole complexes were obtained by using the following purchased chemicals without further purification: Iron(II)Bromide (FeBr_2_) (98%) from Sigma-Aldrich (St. Louis, MO, USA); L-ascorbic acid (>99%) from Carl Roth and 4-Amino-1,2,4-Triazole (99%) purchased from Thermo Scientific (Waltham, MA, USA).

### Synthesis of [Fe(atrz)$_{3}]$Br_2_

To obtain the complex [Fe(atrz)_3_]Br_2_ a modified synthesis based on the procedure by Pidrahita-Bello et al. was performed^48^. At first, 638.32 mg (2.96 mmol) of FeBr_2_ were dissolved in 2.5 mL of deionized H_2_O with 60 mg of L-ascorbic acid. This solution was added to a separate solution of 745.79 mg (8.87 mmol) of 4-Amino-1,2,4-triazole. Subsequently, the solution was stirred for 1 h and a white solid precipitated. This solid was washed three times with 10 ml of ethanol and centrifugated at 5000 rpm (2370 rcf) for 10 min. During the washing process a colour change from white to violet was observed and 1.19 g of the product were obtained (yield: 86%). The corresponding measured spectral data for mid- and far-infrared (FIR) (in cm^-1^) are: 3475(s), 3413(s), 3288(s), 3226(s), 3193(s), 3089(s), 2991(s), 2750(w), 2023(w), 1614(s), 1541(s), 1475(w), 1388(m), 1367(m), 1340(w), 1276(w), 1213(s), 1087(s), 1026(s), 879(m), 690(m), 615(s). Hereby, s means strong, w weak and m medium, respectively, and describing the strength of the absorption bands.

### Characterization

**Figure 1:**
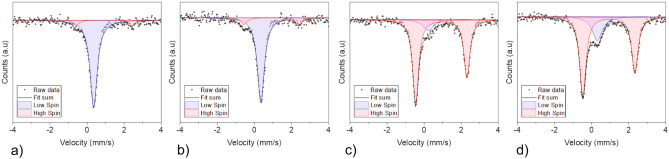
Mössbauer spectra showing the absorption of the high and low-spin states at (**a**) room temperature (20 ^∘^C), (**b**) at 30 ^∘^C, (**c**) after heating to 57 ^∘^C and (**d**) after cooling to 30°. The spectra are taken in transmission configuration as function of the modulation of the gamma ray emitter given in mm/s.

The Iron(II) triazole complex was chosen for the experiments in this work because it shows significant and characteristic changes due to the SCO effect. A measurement based on Mössbauer spectroscopy was performed to obtain the switching behaviour and shows additionally the reversibility of this process. The change of the spectral response is shown in Fig. 1.

Mössbauer spectroscopy uses gamma rays to excite atomic nuclei. Hereby, the absorbed energy differ due to the different spin states of the Fe nuclei within the chemical composition. The colored areas in Fig. 1 represents the absorption of a single spin state. The integral of each colored area corresponds to the amount of nuclei which are in the high or low-spin state. The measurement shows that the spin states are predominantly changing from low-spin (at 20 ^∘^C and 30 ^∘^C) to high-spin (at 57 ^∘^C). The material shows a hysteresis at 30 ^∘^because two spin-states are allowed at this temperature depending if the material is heated or cooled. The corresponding values of the different spin states are summarized in Table 1.

**Table 1:** Amount of the different spin states correlating to the temperatures used for Mössbauer spectroscopy shown in Fig. 1.

Temperature (^∘^C)	High spin state (%)	Low spin state (%)
20	8.3	91.7
30 (heating)	13.5	86.8
57	87.2	12.8
30 (cooling)	77.7	22.3

**Figure 2:**
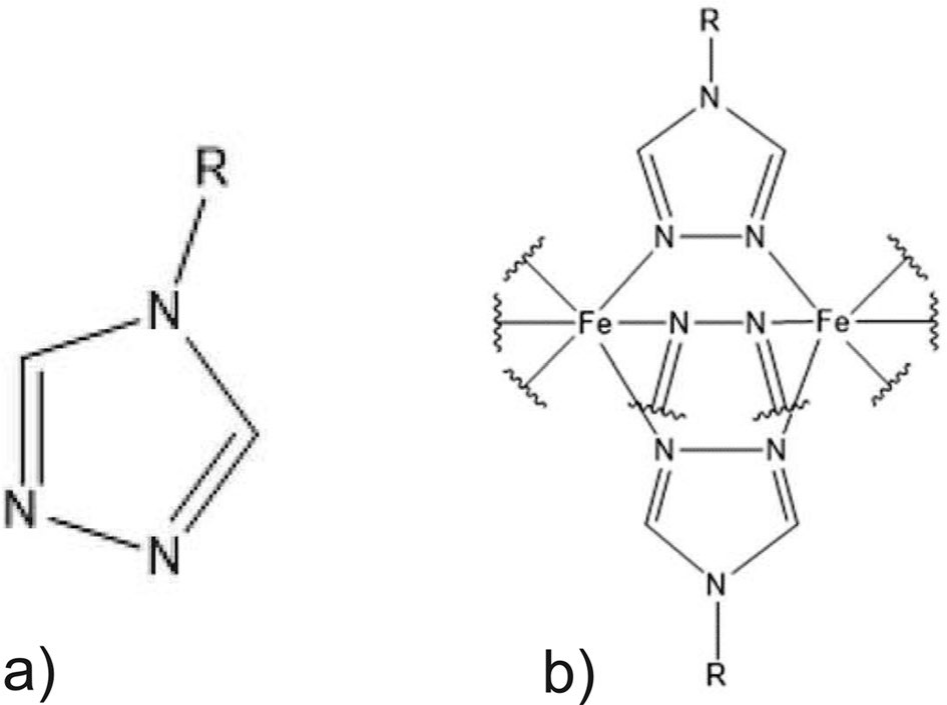
(**a**) Triazole ligand substitued at the 4-position and (**b**) schematic illustration of the one-dimensional coordination chains. R refers to any organic rest at the 4-position.^33^.

The obtained data from Mössbauer and IR-spectroscopy as well as the characteristic purple color of the complex at room temperature are correlating well with the literature^49,50^. The structure of the Iron(II) complex is based on an infinite one-dimensional chain as depicted in Fig. 2.

## Methods

**Figure 3:**
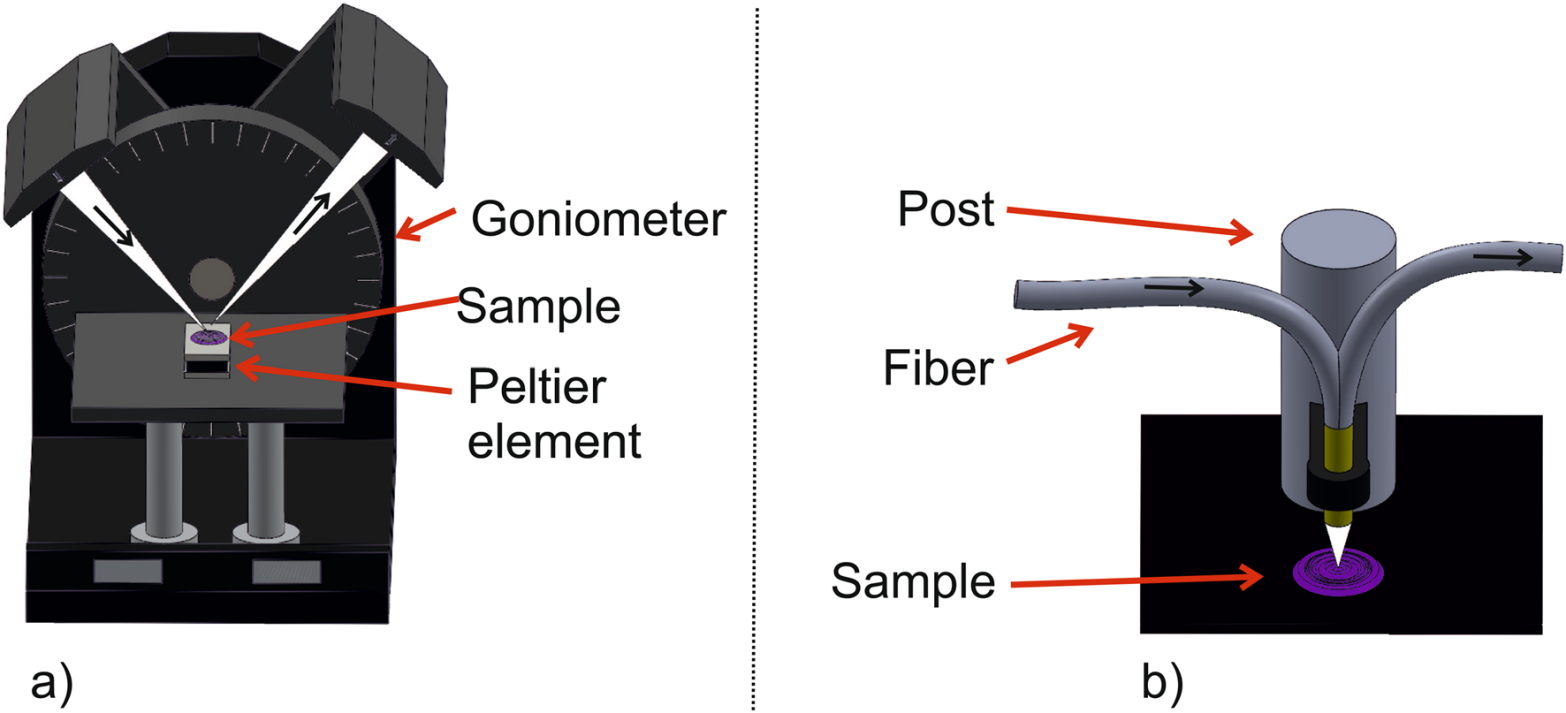
Setups used to characterize molecular switches. (**a**) The goniometer was used to investigate the response of thermal changes and an applied magnetic field. (**b**) The fiber based setup was designed for humidity measurements within a climate chamber.

As an example material demonstrate the potential of molecular switches we used the iron based triazole complex and characterized the relevant optical switching properties. The employed measurement setups are shown in Fig. 3.

A goniometer (Fig. 3a) was used in combination with a fiber coupled white light source (MBB1F1, Thorlabs GmbH, Germany) and a Peltier element (QuickCool, Quick-Ohm Küpper & Co. GmbH, Germany) to illuminate and heat the material and a fiber coupled spectrometer (AVASPEC-ULS2048XL-EVO-RS, Avantes B.V., Netherland) was employed to evaluate the optical transmission. This setup in particular was used to investigate the SCO effect induced by variation of temperature and magnetic field to evaluate the optical transmission. For the measurements, the white-light LED was connected to one arm of the goniometer and illuminated a thin layer of the molecular switches placed on the Peltier element. The reflected and scattered light was guided through an additional fiber, which was connected to the second arm of the goniometer leading towards the spectrometer. The spectrum of the reflected light was monitored continously during systematic variation of the environmental parameters. The setup shown in Fig. 3 b) was used to investigate the effect of a variation of environmental humidity within a climate chamber (605-02, Memmert GmbH + Co. KG, Germany) which can vary the temperature and humidity in a range from 10 up to 95 ^∘^C and 10%  to 98% relative humidity (rh), respectively. Hereby, a special 1:2 fiber splitter (SPLIT400-UV-VIS, Ocean Insight) with a core diameter of 400 $\upmu$m was employed. One end of the fiber splitter is connected to a fiber coupled white light LED (MBB1F1, Thorlabs GmbH, Germany) illuminating the sample perpendicular to the surface. The reflected light was collected by the same fiber and directed to the spectrometer. The electronic equipment was placed outside the climate chamber to avoid any damage and the optical fibers were conducted through a sealeable hole enabling monitoring of the material change during the environmental parameters variation.

**Figure 4:**
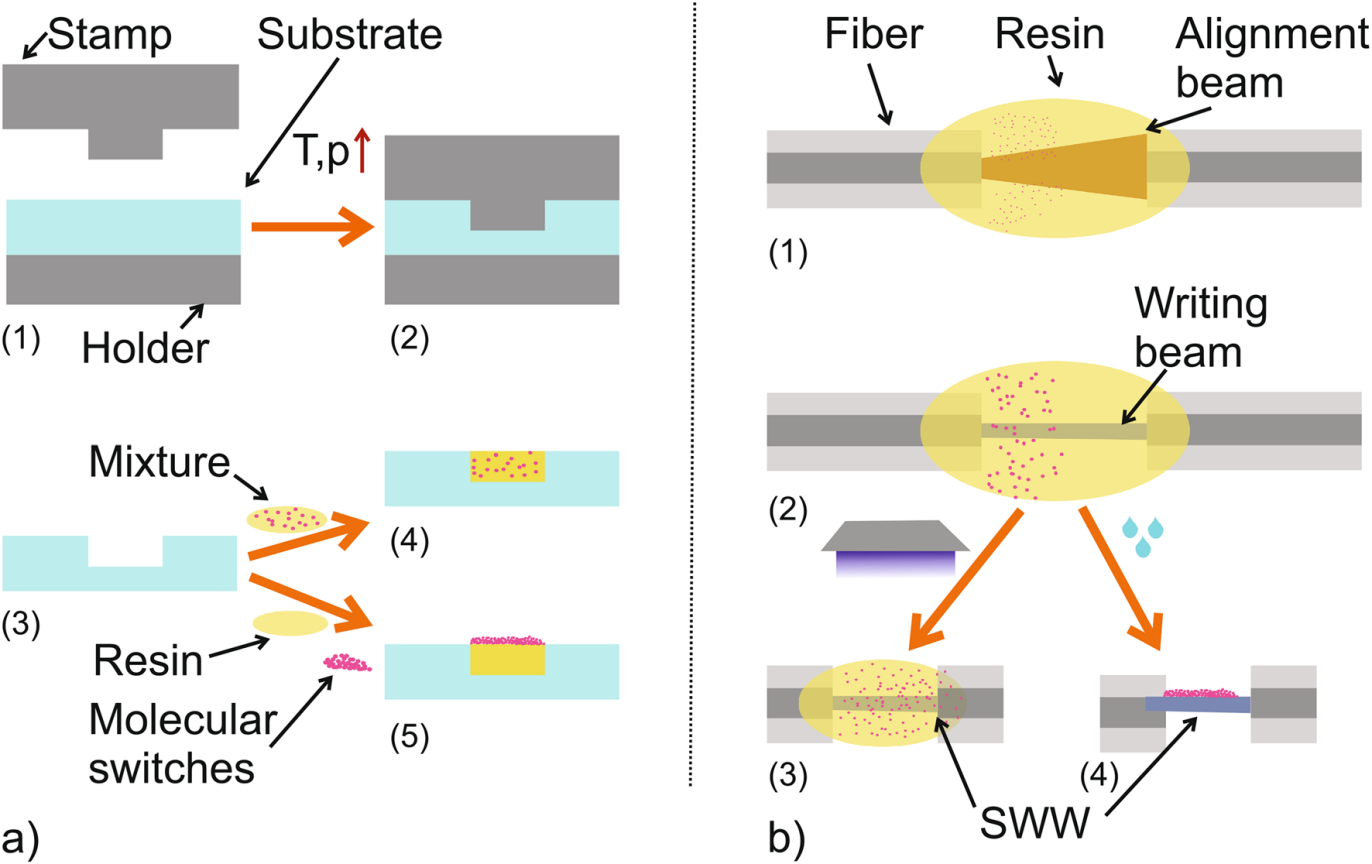
Schemes of the fabrication processes used for realizing the required optical waveguides. (**a**) An hot embossing process was employed to create multi-mode waveguides. Hereby (1) a PMMA foil (Plexiglas XT 99524, ThyssenKrupp, Germany) was placed between a stamp containing the master structure and a planar holder. (2) Applying a temperature slightly above the glass temperature of PMMA ($T_g\approx 120$ ^∘^C) and a pressure of $\approx 35$ bar transferred the structure from the master stamp to the PMMA foil. (3) After the sample cooled down close to room temperature, the pressure was reduced and the sample containing the negative of the master structure was removed. In a final step, the fabricated channel in the PMMA was filled (4) with an epoxy resin (NOA68) mixed with the molecular switches or (5) filled with an epoxy on top of which the molecular switches were applied after the curing process, respectively. (**b**) To investigate single mode structures, SWWs were used. (1) Therefore, an epoxy was applied in between two aligned single mode fibers with a small gap. (2) Subsequently, light with an UV-near wavelength ($\lambda =406$ nm) was illuminated through one fiber starting the curing process of the epoxy locally at the end of the fiber. This, again, increases the refractive index locally, which traps the light and creates a straight waveguide. (3) Finally, the surrounding resin can be cured with UV-flood exposure if the epoxy was already mixed with molecular switches to achieve a distribution of the particle inside the core. (4) Alternatively, the residual resin can be removed using isopropanol enabling free access to the core.

Apart from the detailed analysis of the pure material, we also investigated the behaviour of the molecular switches after embedding them into or onto polymer based optical waveguides. For the characterization, the sample was placed in between two optical fibers for incoupling and outcoupling of the white light which was generated by the same fiber-coupled white light LED used in the experiments described above. Two methods were employed for embedding the molecular switches into waveguides. One concept uses hot-embossed multimode waveguides, imprinted into a 500 $\upmu$m thin PMMA (Polymethylmethacrylate) foil serving as cladding. The embossed channels have rectangular cross-section with dimensions of $\approx$ 250 $\upmu$m $\times$ 250 $\upmu$m. The molecular switches were mixed into an epoxy resin (NOA68, Norland Products, USA) used as core material or were applied on top of the core after the channel was filled. In the latter case, the epoxy resin was cured after floating the channels with electromagnetic radiation at $\lambda =365\,$nm stemming from an UV-lamp (MUV21-254/365, major science, Taiwan). This process is depicted in Fig. 4a).

The second concept to bring the molecular switches in or onto the core of an optical waveguide is the usage of self-written polymer optical waveguides^45,47,51,52^. Hereby, the same epoxy as for the hot embossed waveguides described before is employed. The UV-curable resin was applied in between two optical fibers. Subsequently, the fibers were adjusted towards each other by maximizing the optical transmission using electromagnetic radiation in the visible or infrared wavelength range to avoid curing of the material. Illumination wavelength at $\lambda =\,$406 nm starts the curing process immediately from the end of the fiber. Due to the polymerization process, the refractive index is increased locally which traps the beam and creates a straight connection until reaching the second fiber end. At this point it is possible to cure the surrounding resin with an UV-flood illumination to realize a rigid connection with a still remaining refractive index difference^45^. For our purpose we removed the liquid resin with isopropanol to obtain access to the waveguide core itself. This process is sketched in Fig. 4b).

**Figure 5:**
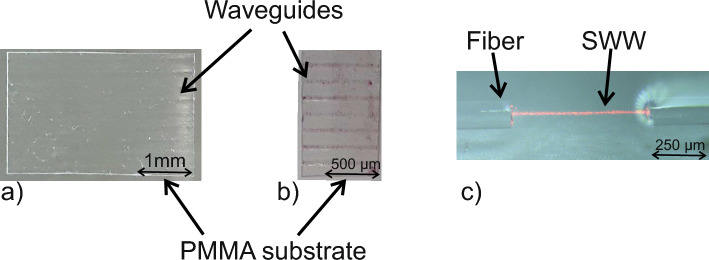
Fabricated waveguides used for the characterization of the molecular switches. Hot embossed waveguide channels filled with NOA68 and (**a**) without and (**b**) with molecular switches on top. (**c**) A self-written waveguided formed between two fibers with a core diameter of 9 µm.

The fabricated waveguides used for the characterization described in Fig. 4 are depicted in Fig. 5.

## Results and discussion

### Bulk material

**Figure 6:**
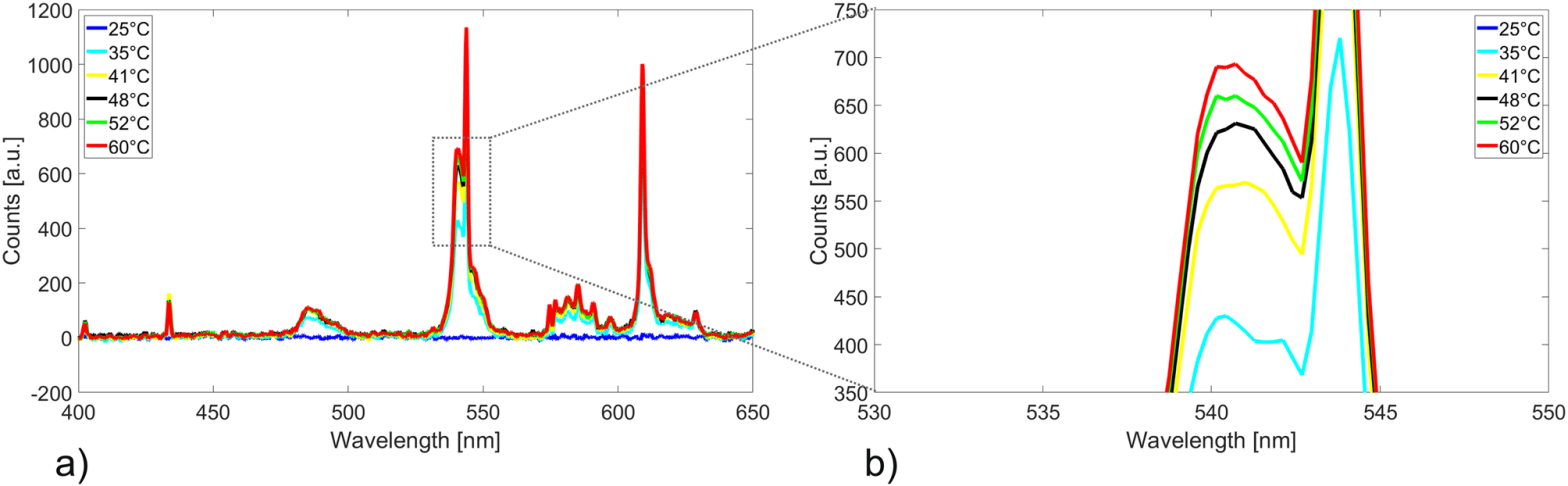
Difference reflection spectra of the molecular switches for (**a**) a wavelength range of 400–850 nm and (**b**) highlighted area for one of the main peaks to depic the clear change with increasing temperature.

Differential measurements were performed by subtracting the spectrum obtained after the variation of a particular parameter from the reference spectrum without variation of that parameter with constant illumination. These difference spectra are directly indicating changes of the absorption characteristics of the material induced by the SCO of the triazole complexes between low-spin (ground state at room temperature) and high-spin states (switched state). The obtained temperature dependant reflection spectra of the pure bulk material is shown in Fig. 6.

**Figure 7:**
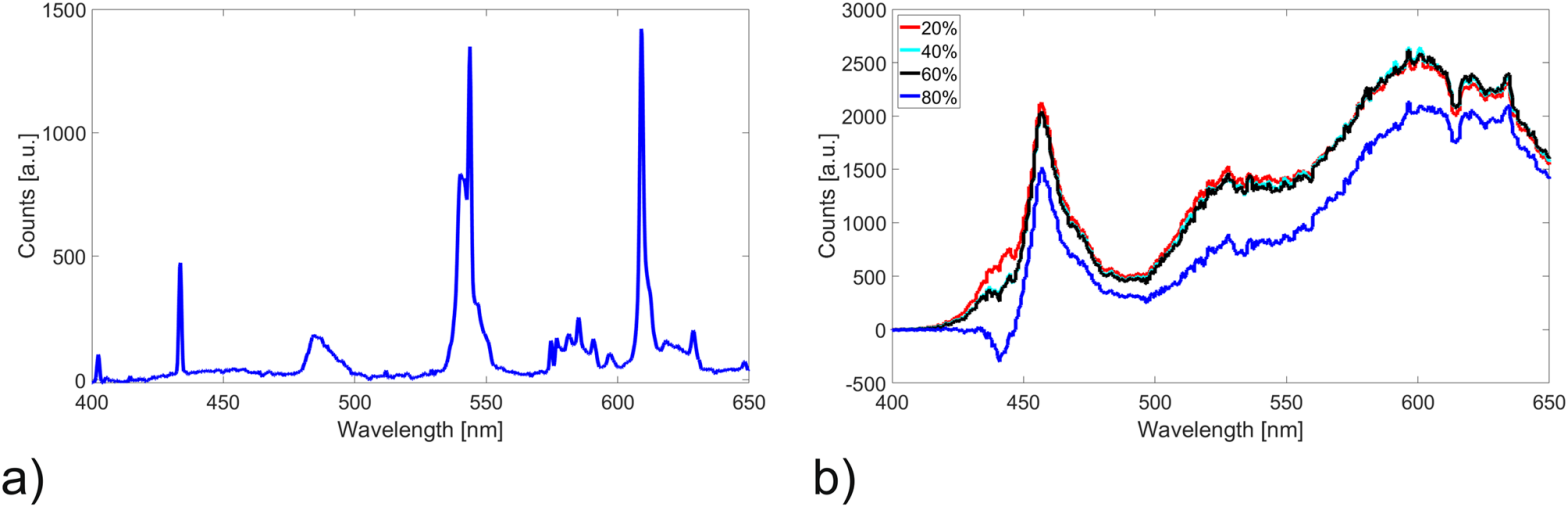
(**a**) Difference reflection spectrum of the molecular switches with applied magnetic field. (**b**) Obtained difference spectrum as function of humidity controlled using the climate chamber.

The investigation of the pure material showed a clear change in the reflection spectra at different specific wavelengths. A peak in that difference spectrum correlates with an increase of the reflected intensity. The enlarged area under the spectrum in Fig. 6b shows the change of the double peaks at $\lambda =540$ nm and $\lambda =543$ nm, respectively, with increasing temperature. These changes of the absorption characteristics of the molecular switches during the transition from low-spin to high-spin state corresponds to a visible color change from violet to white. Similar results were achieved by placing a Nd-based permanent magnet close to the molecular switches. For that measurement, the difference spectrum results from subtracting the spectrum with an applied magnetic field from the one without applied magnetic field. The corresponding spectrum is shown in Fig. 7a. The achieved changes in the spectrum are similar with regard to the wavelength of the peaks from the temperature variation in Fig. 6. The difference in the spectrum could be explained by the characteristic change of color of the material which is caused by the spin crossover phenomenon. This effect is known to also be induced by magnetic fields. Thereby, the thermal hysteresis, which is very close to room temperature could be influenced by the magnetic field^53,54^. In this experiment we were not able to measure the amplitude of the magnetic field applied on the molecular switches. However, we could qualitatively show SCO between different spin states induced by a magnetic field.

The graph in Fig. 7b depicts the measurement performed in the climate chamber by varying the environmental humidity in the range between 10 and 80% rh. In this case, the value of 10% humidity was chosen as ground state which was substracted from the further spectra in this measurement series. The spectral change is not limited to specific wavelength bands, it is rather a modification of the whole absorption characteristic observable even at lower humidity values. The broadening of the whole spectrum might be due to condensation of water on the sample or additional scattering due to a higher humidity. Only at a higher level of $\approx$ 80% an additional shift of the spectra appears. The influence of humidity on the thermal hysteresis of the SCO phenomenon of triazole complexes is well known and documented^38^. It was shown that a higher amount of water especially in amino-triazole complexes results in lower transition temperatures^55^. This means that contact with water and therefore increased humidity promotes the spin transition towards the high spin state at ambient temperature. This explains the difference in the case of the spectrum shown in Fig. 7 and proves that it is caused by the SCO.

### Switching behaviour of triazole complexes integrated in and onto polymer waveguides

The triazole complexes can also be pepared as particles, as described above, and incorporated in or onto optical waveguides. Therefore, the microcrystalline molecular switches were mixed with the epoxy resin NOA68 and processed as described in Fig. 4. Hereby, the amount of the applied Iron(II) complex was varied between 0.5 and 2.0 wt% and its distribution inside the core material was assumed homogenous after the mixing. A higher amount of particles did not lead to an increasing sensitivity, whether applied in or onto the waveguides. The signal decreased if more particles were embedded into the core material due to higher scattering losses. Hot embossing was used to fabricate multi mode waveguides whereas the SWW technique enables manufacturing of single and few mode waveguides, respectively. In general, implementing the molecular switches into the SWW, leads to high scattering losses which is not advantageous for the writing of the SWWs. Therefore, the investigation here were performed by placing the particles on the surface of written few mode SWW structures. Thus, in this work the particles were located both inside the multi mode waveguides and on the core of the single mode SWWs.

#### Molecular switches in and onto multimode waveguides

The waveguides used for this experiment had a cross-section of $\approx$ 250 $\upmu$m $\times$ 250 $\upmu$m determined by the stamp design. These dimensions enable light guiding in the waveguide even with scattering particles inside.

**Figure 8:**
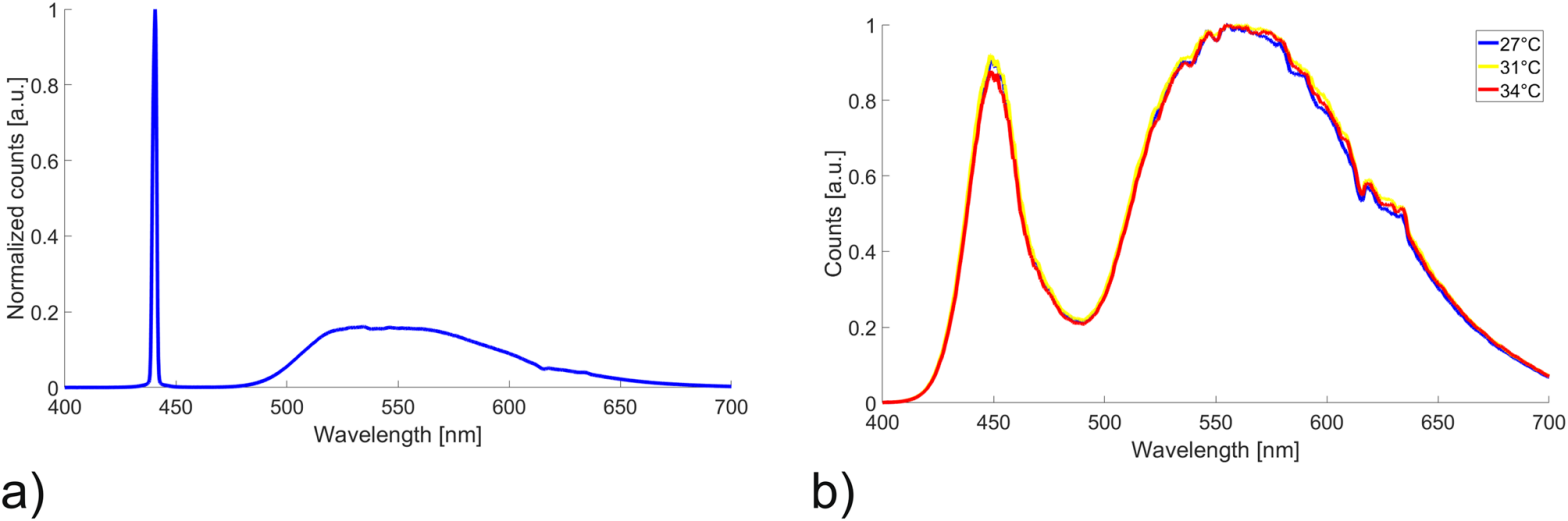
Transmission spectrum of (**a**) radiation from a white light LED through an hot embossed waveguide and (**b**) during the heating process with applied molecular switches inside the core of the multi mode waveguide.

The spectrum of the white light LED guided through a waveguide without additional particles is shown in Fig. 8a and depicts one intense peak in the near-UV range and a broad peak at higher wavelengths which is common for LED based white light sources. For waveguides with integrated molecular switches, spectra as depicted in Fig. 8b were measured. The main differences in the two spectra are the notably attenuated and broadened near-UV peaks which depend on the length of the polymer waveguide due to high absorption and scattering losses compared to radiation at longer wavelength. The effect of embedded molecular switches observed in the spectral range between approximately 615–635 nm and 540–543 nm, respectively. Here, a slightly shift in the transmission is noticeable which correlates with the significant spectral changes of the bulk material depicted in Fig. 6. The spectral changes with particles embedded in the waveguides are considerably smaller due to lower concentration of the molecular switches.

#### Molecular switches in and onto few mode waveguides

**Figure 9:**
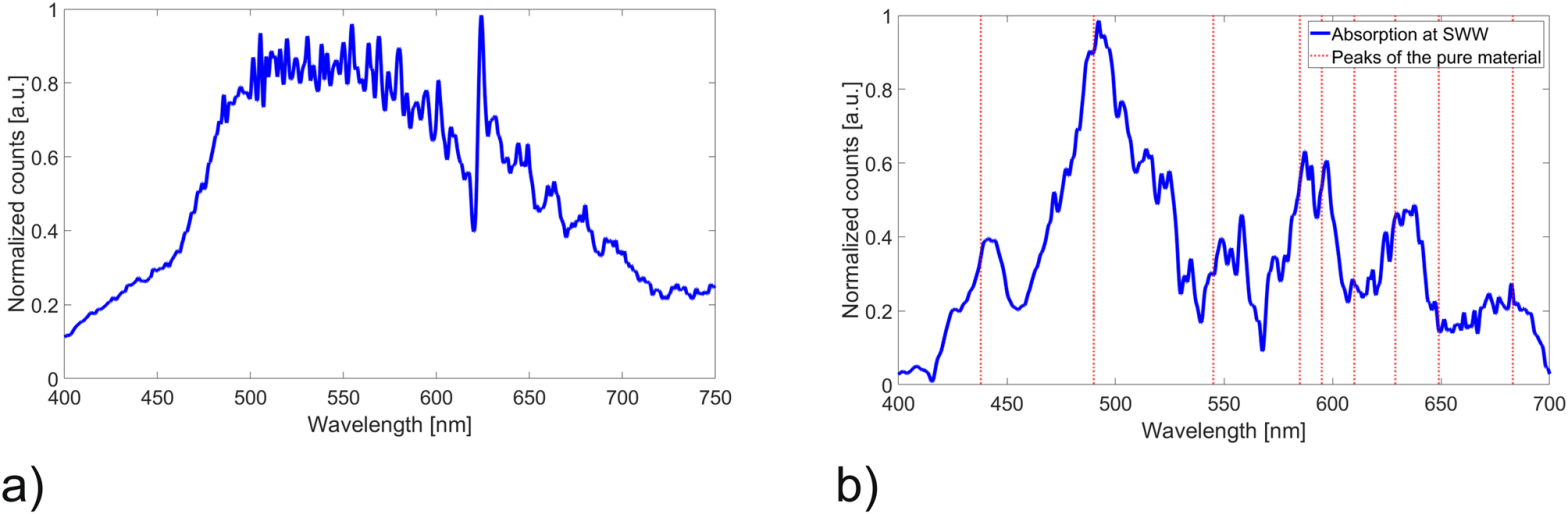
(**a**) Transmission spectrum of the laser-driven white light plasma source through a single mode SWW with removed cladding and (**b**) difference transmission spectrum with applied molecular switches heated to 34 ^∘^C.

SWWs as depicted in Fig. 4b were prepared and layers of molecular triazole complexes were deposited onto the waveguide surface as described above. The advantage of using SWW are the processability enabling fabrication of single-mode waveguides and that the core is accessible by removing the uncured resin with isopropanol. The applied molecular switches on the surface of the waveguide are able to interact with the evanescent field of the transmitted light. To create the SWWs a standard single-mode fiber with a core diameter of 9 $\upmu$m was used. The molecular switches were applied on the core and subsequently heated with a Peltier element placed below the structure. The obtained results are shown in Fig. 9b.

For this experiment a light source with a higher optical power was required. Therefore a laser-driven white light plasma source (Energetiq Technology, EQ-99X, USA) was employed. The transmission spectrum through an SWW without applied molecular switches is depicted in Fig. 9a. The obtained results with applied material are shown as normalized absolute values of the difference spectra with and without molecular switches in Fig. 9b. Some of the peaks correlate well with the absorption characteristics of the pure material others are more difficult to be distinguish from the background noise originating from the light source. The shift of the obtained peaks compared to the pure material decreases at higher wavelengths. A reason for this behaviour could be that a thin film of liquid resin remains around the SWW after removing of the residual resin material. The applied molecular switches are in direct contact with the remaining resin which might lead to a shift at shorter wavelength were the absorption of the monomer is higher compared to the case of larger wavelengths. In any case, a clear correlation between the applied molecular switches and the spectral variation induced by the SCO is observable.

The measurements from the bulk material where the SCO was detected by applying a magnetic field were repeated with the molecular switches in and onto the optical waveguides. The effect could not be observed in this configuration which is probably due to the significantly lower concentration of particles affected by the magnetic field on the waveguide compared to the high concentration in the bulk material.

## Conclusion

In this work, the switching behaviour of an iron triazole complexe was analyzed as function of several external parameters such as humidity, temperature and magnetic fields. The SCO occurs due to the transition of low-spin states to high-spin states induced by the mentioned parameters. As an example material, iron triazole complexes were employed. The investigations were conducted by using pure bulk material synthesized according to Pidrhita-Bello et al.^48^ incorporated as micro particles inside the optical waveguide cores or deposited on their surface. Especially the pure material showed significant changes in the reflected spectrum during variation of external parameters. To demonstrate the switching behaviour, difference spectra of the molecular switches in the high-spin state populated through heating or an applied magnetic field were recorded and compared. The results of the humidity measurement also display a significant change in the spectra, however might be affected by condensed water. The process itself is reversible and sensitive to various parameters. The molecular switches have additionally been integrated into optical waveguides by mixing them with an optical expoxy resin. Hereby, the used iron triazole complex keeps it switching behaviour after incorporating within the epoxy and the subsequent UV-curing step. Using such structures, it was possible to detect variations in the transmitted optical signal of the waveguides based on the switching process, however, the signal size was lower compared to the reflection spectra of the pure material. In the next steps, we will investigate the influence of the size of the particles which ultimately influence scattering strength of the stucture and can be used to control the signal strength. Recent results enable an embedding of the particles into a polymer matrix leading to a change from a transparent milky-white polymer to a high absorbing violet one. This behaviour is highly promising towards further optical applications such as sensing of environmental parameters.

## Data Availability

All data generated or analyzed during this study are included in this article. The raw data are available upon reasonable request.
